# Co-prevalence of human Papillomaviruses (HPV) and Epstein–Barr virus (EBV) in healthy blood donors from diverse nationalities in Qatar

**DOI:** 10.1186/s12935-020-01190-2

**Published:** 2020-04-03

**Authors:** Ishita Gupta, Gheyath K. Nasrallah, Anju Sharma, Ayesha Jabeen, Maria K. Smatti, Hamda A. Al-Thawadi, Ali A. Sultan, Moussa Alkhalaf, Semir Vranic, Ala-Eddin Al Moustafa

**Affiliations:** 1 College of Medicine, QU Health, Qatar University, Doha, Qatar; 2 Biomedical Research Centre, Qatar University, Doha, Qatar; 3 Weill-Cornell Medicine-Qatar, Doha, Qatar; 4 Faculty of Medicine, Kuwait University, Kuwait City, Kuwait

**Keywords:** HPV, EBV, Healthy blood donors, Qatar

## Abstract

**Background:**

Infections by both human oncoviruses, human Papillomaviruses (HPV) and Epstein–Barr virus (EBV) are very common in the adult human population and are associated with various malignancies. While HPV is generally transmitted sexually or via skin-to-skin contact, EBV is frequently transmitted by oral secretions, blood transfusions and organ transplants. This study aims to determine the prevalence and circulating genotypes of HPV and EBV in healthy blood donors in Qatar.

**Methods:**

We explored the co-prevalence of high-risk HPVs and EBV in 378 males and only 7 females blood donors of different nationalities (mainly from Qatar, Egypt, Syria, Jordan, Pakistan, and India) residing in Qatar, using polymerase chain reaction (PCR). DNA was extracted from the buffy coat and genotyping was performed using PCR and nested-PCR targeting E6 and E7 as well as LMP-1 of HPV and EBV, respectively.

**Results:**

We found that from the total number of 385 cases of healthy blood donors studied, 54.8% and 61% of the samples are HPVs and EBV positive, respectively. Additionally, our data revealed that the co-presence of both high-risk HPVs and EBV is 40.4% of the total samples. More significantly, this study pointed out for the first time that the most frequent high-risk HPV types in Qatar are 59 (54.8%), 31 (53.7%), 52 (49.1%), 51 (48.6%), 58 (47%) and 35 (45.5%), while the most commonly expressed low-risk HPV types are 53 (50.6%), 11 (45.5), 73 (41.7%) and 6 (41.3%), with all the cases showing multiple HPVs infection.

**Conclusion:**

In this study, we demonstrated for the first time that HPV and EBV are commonly co-present in healthy blood donors in Qatar. On the other hand, it is important to highlight that these oncoviruses can also be co-present in several types of human cancers where they can cooperate in the initiation and/or progression of these cancers. Therefore, more studies regarding the co-presence of these oncoviruses and their interaction are necessary to understand their cooperative role in human diseases.

## Background

Human Papillomaviruses (HPVs) and Epstein–Barr virus (EBV) are human oncoviruses that can co-exist together. HPVs are small, double-stranded DNA viruses which tend to infect cutaneous and mucosal epithelial tissues of the ano-genital tract [1]. HPVs are categorized into high-risk or low-risk. Infections due to low-risk HPV subtypes (mostly, HPVs-6 and 11) are commonly self-limiting diseases resulting in the proliferation of epithelial cells that manifests as skin or genital warts or skin papilloma [2, 3]. On the other hand, high-risk HPV subtypes (16, 18, 31, 33, 35, 39, 45, 51, 52, 56, 58, 59 and 68) are associated with the development of several types of human carcinomas [2, 3]. In particular, cancers developing in the cervix, ano-genital tract as well as oropharynx region are frequently correlated with chronic high-risk HPV infection [4, 5]. While HPV infections are eradicated by the immune system, persistent chronic infections associated with high expression levels of E6/E7 (viral oncogenes) as well as accretion of cellular mutations may cause alterations in *TP53* and *RB* expressions, which are known as tumor suppressor genes [6]. Additionally, it has been reported that high-risk HPVs (E5 and E6/E7) oncoproteins, can enhance cancer progression of human carcinomas via the initiation of the epithelial-mesenchymal transition (EMT) which is a hallmark of cancer metastasis [7–9]. On the other hand, it is important to note that HPVs transmissions may be enhanced by the presence of rough or macerated epithelial surfaces [10]. Several studies have demonstrated that HPV DNA can be present in circulating blood, including peripheral blood mononuclear cells (PBMCs), sera, plasma, and arterial cord blood [11–15].

Human herpesvirus 4, commonly known as Epstein–Barr virus is a DNA lymphotropic herpesvirus that causes infectious mononucleosis [16]. EBV comprises of a 172 kbp double-stranded DNA genome encoding for around 85 genes [17, 18]. There are two EBV genotypes, Type 1 and Type 2 [19]. Various viral oncogenes such as EBV-encoded nuclear antigens (EBNA [1–3]) and the latent membrane proteins (LMP [1, 2]) are encoded by the EBV [20]. LMP-1 is a 356-amino acid protein [21] and is involved in signal transduction and cell survival [18]. Since LMP1 regulates cell growth, protects cells from apoptosis, promotes cell motility, and angiogenesis, it is considered as the principal EBV-encoded oncogenic protein [22]. LMP-1 variants are divided into 7 core groups including B95-8, Alaskan, China 1, China 2, Med+ , Med− , and NC [18, 23, 24]. LMP1 can stimulate several signaling pathways especially those involved in the induction of EMT such as NF-κB, PI3K/Akt, and MAPK [7]. The other EBV onco-protein, LMP2A [25] regulates the invasive/migratory ability and stimulates changes in EMT-like cellular biomarkers [26] by targeting the rapamycin (mTOR) pathway [22]. On the other hand, EBNA1, a key product of EBV, is involved in the replication and maintenance of the EBV genome [27] and regulates EMT through the de-regulation of SLUG, SNAIL, TCF8/ZEB1, vimentin, occludins-1, as well as E-cadherin [9]. EBV is primarily transmitted through the oral route; however, blood transfusions and organ transplantations are also other possible transmission routes of EBV [28–30].

Several recent studies have demonstrated that the co-presence of high-risk HPVs and EBV mRNA in various human malignancies including cervical, breast and oral cancers, indicating their active role in cancer initiation and metastatic progression via the acceleration of EMT through numerous common signaling pathways [11, 31].

In light of these findings, the objective of this study is to explore the presence of HPV and EBV DNA in the blood of healthy individuals in the Middle East region, more specifically in Qatar.

## Materials and methods

### Sample collection and ethical approval

A total of 385 whole blood samples (139 from Qataris and 246 from other nationalities) were collected in EDTA tubes from healthy donors at the Blood Donor Unit of Hamad Medical Corporation (HMC) over a period of 1 year (September 2014–September 2015) as previously described [32]. Blood samples were handled and stored following standard safety procedures and guidelines. This study was approved by HMC-Institutional Review Board (HMC-IRB #14292/14) and Qatar University IRB (QU-IRB 518-EA/15) [32].

### DNA extraction

DNA was extracted from PMNCs (buffy coat samples) suspended in 200 μl of PBS using Qiagen kit following the manufacturer’s instructions (Qiagen, Germany) as previously described [32]. The concentration and purity of all extracted DNA samples were measured using NanoQuant microplate reader (Tecan, Switzerland). Extracted DNA samples were then stored at − 20 °C for further testing.

### HPV and EBV detection

Twenty-five nm of purified genomic DNA (Qiagen, Germany), from each sample, was analyzed for HPV and EBV by polymerase chain reaction (PCR) using specific primers as follows:

The E6/E7 and L1 region of HPV types; GP5/6, 6, 11, 16, 18, 26, 31, 33, 35, 39, 45, 51, 52, 53, 56, 58, 59, 66, 68, 73 and 82 were amplified (Table 1), while, primers for GAPDH gene (Forward Primer: 5′-GAAGGC-CATGCCAGTGAGCT-3′ and Reverse Primer: 5′-CCGGGAAACTGTGGCGTGAT-3′) were used as an internal control.

**Table 1:** The specific primer sets for HPV used for polymerase chain reaction (PCR) amplification

HPV type	Forward primer (5′-3′)	Reverse primer (5′-3′)	Ta (°C)
E6/E7 region			
16	ATGCATGGAGATACACCTACATTGCAT	GTTTCTGAGAACAGATGGGGCACAC	61
18	GCTTTGAGGATCCAACACGG	TGCAGCACGAATGGCACTGG	61
31	GGGCTCATTTGGAATCGTGTG	AACCATTGCATCCCGTCCCC	61
33	TGAGGATGAAGGCTTGGACC	TGACACATAAACGAACTGTG	61
35	CTATTGACGGTCCAGCT	TACACACAGACGTAGTGTCG	61
45	CCCACGCGAACCACAG	TCTAAGGTCCTCTGCCGAGC	46.3
51	TACGTGTTACAGAATTGAAG	AACCAGGCTTAGTTCGCCCATT	46.3
52	GCAGAACAAGCCACAAGCAA	TAGAGTACGAAGGTCCGTCG	60
58	CGAGGATGAAATAGGCTTGG	ACACAAACGAACCGTGGTGC	40.5
L1 region			
GP5/GP6	TTTGTTACTGTGGTAGATACTAC	GAAAAATAAACTGTAAATCATATT	50.5
6	TGTCCCATCTGCGCACCGAAGAC	CGTACACTGTTTGTGGGCGCTTC	42.5
11	AGTTCCGTAGATGCCAAGGGCA	TGCCTCAGGTGAGGCCCAATGC	42.5
26	TGGTATACAACGAGTGTCAGCTCC	GGGGCAATGATGGCCATGTCG	40.5
39	TGTGCAGTACCAGTGACGGATCG	ATTTTTGGCGTTGTGACTCTGTG	40.5
53	TTGTTCAGTGTACGGGGCTAGC	GTGACGCCATTGCAGTTATCGCCT	43.3
56	CTGGGCACTAGGTCAAAGCCTGCT	CAACCACGCGTAAAAGCACTCAT	53.8
59	AGACACCGTTACATGAGCTGCT	TCATTCTCGGAGTCGGAGTCAG	43.3
66	TGCGGTAGTATCCTTGGGCAGTG	TACAATAAGGGCCACACGCCAA	46.3
68	GTCAAAAAGACGCCCCTGCACCTA	CACACCTTAGGGTAGGGCTACAA	48.8
73	GGGGTGGGCAAAGGTAGGTAGC	ACAATCCAGGGGCCTCTGGTCCGA	48.8
82	TGTCCGTGGACACCTGCGACCA	GTAGTTAAAGGTGATGTGGCAACC	48.8

On the other hand, a nested PCR of the LMP-1 gene was done as previously described [32]. In the first round of amplification, primers A1 (5′-AGTCATAGTAGCTTAGCTGAA-3′) and A2 (5′-CCATGGACAACGACACAGT -3′) amplified a fragment of 602 bp covering the C-terminus of LMP-1 gene. In the second round of amplification, primers B1 (5′-AGTCATAGTAGCTTAGCTGAA-3′) and B2 (5′-CAGTGATGAACACCACCACG-3′) amplified a 587 bp fragment. The annealing temperature for the LMP-1 gene was 53 °C.

Amplification conditions included an initial heat activation step of 15 min at 95 °C; 40 cycles of PCR amplifications at 95 °C for 30 s, annealing (Table 1) for 30 s in addition to 1 min at 72 °C; and a final extension step of 72 °C for 10 min. Analysis was performed as previously described by our group [33, 34]. Amplified products were analyzed by 1.5% agarose gel electrophoresis and visualized using iBrightCL1000 Imaging System (ThermoFisher). In each experiment, negative control (MDA-MB-453 cell line [35] and sterile water instead of DNA) and positive control (such as Hela cell line for L1 region [36] and normal oral epithelial (NOE) cell line transfected with E6/E7 of HPV type 16 for E6/E7 region [37]) were used.

### Statistical analysis

To determine the relation significance between variable ratios, Chi square test was used. Results with p-value < 0.05 were considered statistically significant. The analysis was performed using Statistical Package for Social Sciences (SPSS) version 25 software (IBM SPSS).

## Results

### Demographic data and main findings

Of the total 385 blood samples analyzed in the present study, 378 samples (98.1%) were from males and seven samples (1.8%) from females. The majority of the samples were obtained from non-Qatari residents (66.2%), and the rest were from Qatari individuals (33.7%). The age of participants ranged between 19 and 68 years, (37.12 ± 9.3 years). Based on the study conducted previously [32], of the total 385 blood samples, 235 (61%) were positive for EBV; Table 2 summarizes the demographic data of the studied population. The present investigation revealed that 60.5% and 47.3% are positive for low-risk and high-risk HPVs, respectively. In parallel, we were able to show that EBV and HPVs are co-present in 40.4% and 50.6% for high-risk and low-risk HPVs, respectively.

**Table 2:** Demographic data of the subjects positive (235) and negative (150) for EBV

	EBV positive (235)	EBV negative (150)
Category	Total no. (%)	Total no. (%)
Nationality & gender		
Qatari	102 (43.4%)	28 (18.66%)
Male	101	27
Female	1	1
Non-Qatari	133 (56.5%)	122 (81.33%)
Male	131	119
Female	2	3
Age group		
20–39	52 (22.12%)	46 (30.66%)
30–39	95 (40.4%)	63 (42%)
40–49	60 (25.5%)	34 (22.66%)
50–59	28 (11.91%)	7 (4.66%)

### Prevalence of low-risk HPVs in healthy blood donors in Qatar

We herein explored the presence of low-risk HPVs in healthy blood donors from Qatar, in both EBV positive and negative groups. Table 3 summarizes the prevalence of low-risk HPVs in healthy blood donors, based on our PCR analysis using specific primers for LMP1 as well as E6/E7 genes of EBV and HPVs, respectively (Materials and Methods Section). It is worth noting that using GP5+/6+ primer set is a highly sensitive specific method for HPV DNA detection [38] as it targets L1 conserved regions of the viral genome; thus, allowing detection of a comprehensive variety of HPV genotypes [39, 40]. We found that GP5/GP6 was positive for more than 60% of the samples and hence, further analysis was performed to determine HPV subtypes.

**Table 3:** Prevalence of low-risk HPVs in healthy blood donors from Qatar

Samples	No. of cases	Low-risk HPV types (%)			
		6	11	53	73
EBV (+)	235	41.3	45.5	50.6	41.7
EBV (−)	150	76	63.3	54	75.3
Total	385	54.8	52.5	51.9	54.8

**Fig. 1:**
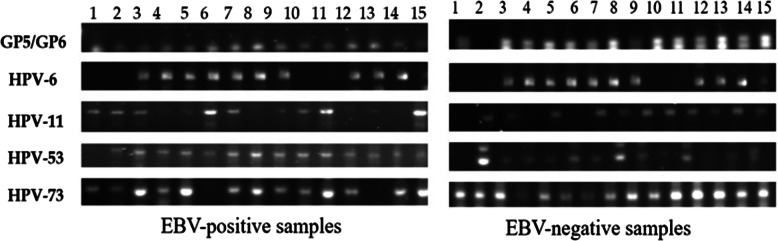
Representative PCR reactions for low-risk HPV-subtypes in 15 different EBV-positive and EBV-negative healthy blood donors

Analysis revealed that of the total 385 samples, 235 were positive and 150 were negative for EBV. On the other hand, 233 of the total samples (60.5%) were positive for low-risk HPVs, and all of the positive cases were infected with more than one type of low-risk HPV (Fig. 1). This number represents 50.6% of the 235 EBV-positive samples, and 76% of the 150 EBV-negative samples. Moreover, data revealed that the most prevalent low risk HPV types were HPV-6 and 73 (both at 54.8%), followed closely by HPV-11 (52.5%) and HPV-53 (51.9%) (Table 3).

### Prevalence of high-risk HPVs in healthy blood donors in Qatar

**Fig. 2:**
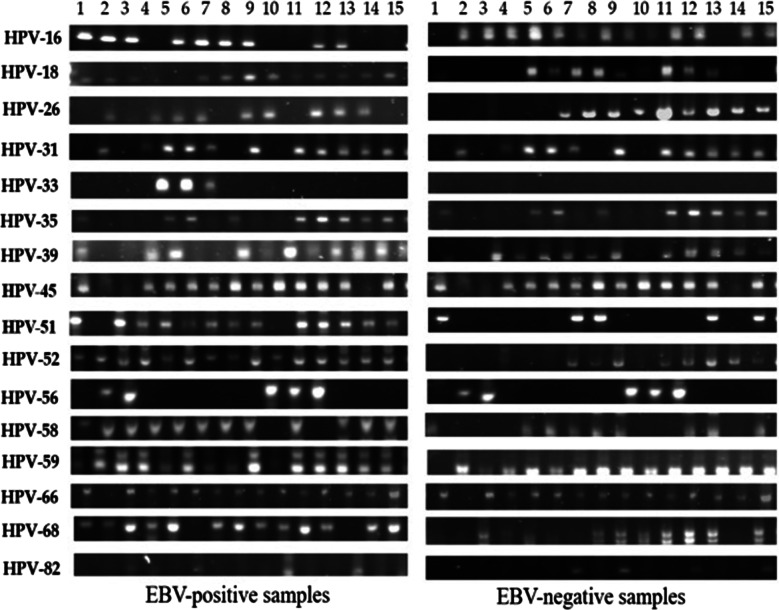
Representative PCR reactions for high-risk HPV-subtypes in 15 different EBV-positive and EBV-negative samples of healthy blood donors

We further investigated the co-presence of high-risk HPVs and EBV in our samples by PCR using specific primers for E6/E7 as well as LMP1 genes of HPVs and EBV, respectively (Table 4). Analysis revealed that 182 of the total number of samples were positive for high-risk HPVs (47.3%), representing 40.4% of the 235 EBV-positive and 58% of the 150 EBV-negative samples. Again, all cases were infected with more than one high-risk HPV type (Fig. 2). Moreover, data revealed that the most prevalent high-risk HPV types in EBV-positive donors were HPV-59 (54.8%), HPV-31 (53.8%), HPV-52 (49.1%), HPV-51 (48.6%), HPV-58 (47%), HPV-35 (45.4%), HPV-26 (43.4%), HPV-16 (37.9%) and HPV-18 (36.1%).

**Table 4:** Prevalence of high-risk HPVs in samples from healthy blood donors in Qatar

Samples	No. of cases	High-risk HPV types (%)															
		16	18	26	31	33	35	39	45	51	52	56	58	59	66	68	82
EBV (+)	235	35.7	34.5	32.8	39.6	4.6	35.3	32.8	30.6	36.2	37.9	27.7	37.4	40.4	29.4	25.5	20.8
EBV (−)	150	41.3	38.6	60	76	0	61.3	62	56.6	68	66.7	22.6	62	77.3	39.3	71.3	11.3
Total	385	37.9	36.1	43.4	53.8	2.8	45.4	44.2	40.7	48.6	49.1	25.7	47	54.8	33.3	43.3	17.1

### Demographic association of HPV-Subtypes and EBV in Healthy Donors in Qatar

A demographic analysis of the samples from healthy donors by classifying them into Qataris and Non-Qataris showed that, in EBV-negative samples, HPV type 35 is the only highly prevalent subtype in both populations (65.4% and 84%, respectively, p = 0.029). However, in EBV-positive samples, as shown in Table 5, the most prevalent HPV-subtypes include HPV types 52, 56, 66 and 68 (p = 0.02, p = 0.03, p = 0.03, p = 0.02, respectively).

**Table 5:** Association of HPV subtypes in 235 EBV-positive samples from Qatari vs non-Qatari healthy blood donors

QATARIS v/s NON-QATARIS				
Samples	HPV-subtypes (%)			
	52	56	66	68
Qataris (102)	47	37.2	39.2	19.6
Non-Qataris (133)	30.8	24.8	26.3	32.3
p-value	0.022*	0.034*	0.0355*	0.029*

## Discussion

Viral infections are the most common etiologic causes of infection-related cancer agents (~ 15%) [41, 42]. Although these infections predominantly affect developing countries, their frequency cannot be ignored in developed countries [43, 44]. In 38% of virus-related cancers, there is co-presence of HPVs along with EBV [42]. However, these cancers develop after a relatively long latent period (15–40 years) [45]. Viral infections within cells are not limited and their combined oncogenic effects can lead to the onset and/or progression of cancer disease [42, 46].

In the present study, we investigated the co-presence of both low- and high-risk HPVs and EBV in the blood of healthy individuals from the middle east region, more specifically in Qatar. Several previous studies have already shown the occurrence of HPVs in subjects with unidentified HPV-related disease or cancer; viral DNA/RNA being identified in several tissues including sperm cells [47, 48], placental tissue [49, 50] as well as peripheral blood cells [51, 52]; indicating that HPV can disseminate through the bloodstream [13, 47].

Additionally, based on few studies, HPV DNA was shown to circulate in the bloodstream during ephemeral asymptomatic infections [53–55]; however, it was indicated that detection of HPV DNA in blood samples is a useful and potential biomarker of severe HPV-related diseases including cancer. Furthermore, studies have detected HPVs in blood samples of both males and females with non-cancer related HPV urogenital infections. While an earlier study in asymptomatic women with urogenital infections reported HPV positivity in peripheral blood cells [52], another study on male patients’ blood showed HPV DNA detection in semen infection [47]. In the present study, we revealed that the presence of both low-risk and high-risk HPVs is ~ 60.5% and 47.3% in healthy blood donors in a diverse population group from Qatar, respectively. Similar to our study, a study among healthy Australian male donors conducted by Chen et al. revealed the prevalence of 8.3% for HPV infection [51]. Metagenomics analysis using whole-genome shotgun sequencing in 748 samples collected from a cohort of 103 healthy North American human subjects was conducted by the NIH Human Microbiome Project. From each subject, four organs (skin, vagina, mouth, and gut) were used for sample collection. This study in healthy humans, who did not display classical HPV-associated diseases, revealed the overall HPV prevalence of 68.9%; the highest being present in the skin (61.3%), followed by the vagina (41.5%), mouth (30%), and gut (17.3%). The co-existence of multiple HPV types was found in 48.1% of HPV-positive samples [56]. This high prevalence of HPV and coexistence of HPV subtypes in the blood of healthy subjects could be attributed to its presence at multiple body sites, as reported previously [56].

Our study has some limitations: Given that it included a very low number of female participants due to socio-cultural factors and religious/fatalistic barriers, in addition to low levels of hemoglobin in women which is reported as a cause of donor loss [57–60], it is essential to perform other studies of a larger number of cases that may ensure a higher female participation in Qatar in order to clarify gender variations in HPV and EBV distribution in this population. Such studies are essential for the region in order to allow for the selection of the right HPV and EBV vaccines as a necessary preventive tool against several serious diseases associated with these oncoviruses, including cancer. In addition, we cannot anticipate the real clinical impact of the observed frequencies of HPV and EBV in the population of Qatar. However, our group is currently exploring the co-presence of HPVs and EBV in different types of cancers in Qatar including breast, cervical as well as colorectal; analysis in breast and cervical cancers can aid in providing clear data about role of high-risk HPVs and EBV in female cases.

On the other hand, our present study showed for the first time the co-presence of high and low-risk HPVs with EBV in 40.4% and 50.6% of the examined samples, respectively. More specifically, in EBV-negative samples, approximately more than half of the Qataris were positive for low-risk skin HPV-types HPV-6, 11, 53 and 73 (Table 3) that are frequently expressed in asymptomatic infections of normal, healthy skin [61, 62]. The rest were high-risk HPV types that are linked with cancer development. Moreover, the most significantly expressed high-risk HPV subtypes in Qatari healthy donors are in line with previous studies on HPV prevalence in the Middle East [63–65], thus they included HPV-52, 56, 66 and 68 (p = 0.02, p = 0.03, p = 0.03, p = 0.02, respectively, Table 4). Although the percentage of Qatari healthy donors in this study is small, this HPV DNA prevalence in healthy males’ merits further investigation.

## Conclusions

Our study provides evidence that HPV and EBV can be detected and quantified in blood samples of healthy blood donors without any obvious HPV infection, suggesting potential viral persistence at different anatomical sites. This random distribution of HPV subtypes along with EBV, suggests potential facilitative or competitive interactions among these onco-viruses, which has been recently reported by several studies [31, 34, 46, 66]. Since these oncoviruses can be found in various cancers; our group has previously demonstrated that E6/E7 of high-risk HPVs can convert non-invasive cancer cells to invasive phenotypes [22]. Additionally, HPVs genotyping can help select the most relevant HPV vaccine to prevent HPV-associated cancers. However, further studies are required to assess the role of HPV DNA detection in the bloodstream of people with asymptomatic infection to elucidate the underlying mechanisms of the co-presence of HPVs and EBV in healthy donors; especially since vaccines for these onco-viruses are available or are currently under clinical trial [67–69]. This is a vital step as vaccinating individuals can aid in lowering the risk of viral transmission in the common population and ultimately prevent the onset of HPVs- and EBV-related diseases, including cancer. The co-prevalence of HPVs and EBV among healthy blood donors should draw the attention of clinicians, researchers and healthcare workers in Qatar, which could aid in promoting safety practices in health care centers, especially in blood banks and organ transplant centers.

In conclusion, our study revealed that both low-risk and high-risk HPVs are frequently co-present with EBV among healthy blood donors in the heterogeneous population of Qatar. Further observational and clinical studies are required to assess the full relevance of the obtained data.

## Data Availability

All data generated or analyzed during this study are included in this published article [and its additional files].

## Abbreviations

DNA: Deoxyribonucleic acid
EBV: Epstein–Barr virus
EBNA: EBV-encoded nuclear antigens
HPV: Human Papilloma virus
LMP: Latent membrane protein
PCR: Polymerase chain reaction
