# Correction to: Co-prevalence of human Papillomaviruses (HPV) and Epstein–Barr virus (EBV) in healthy blood donors from diverse nationalities in Qatar

**DOI:** 10.1186/s12935-022-02459-4

**Published:** 2022-01-29

**Authors:** Ishita Gupta, Gheyath K. Nasrallah, Anju Sharma, Ayesha Jabeen, Maria K. Smatti, Hamda A. Al-Thawadi, Ali A. Sultan, Moussa Alkhalaf, Semir Vranic, Ala-Eddin Al Moustafa

**Affiliations:** 1 College of Medicine, QU Health, Qatar University, Doha, Qatar; 2 Biomedical Research Centre, Qatar University, Doha, Qatar; 3 Weill-Cornell Medicine-Qatar, Doha, Qatar; 4 Faculty of Medicine, Kuwait University, Kuwait City, Kuwait

## Correction to: Cancer Cell Int (2020) 20:107 https://doi.org/10.1186/s12935-020-01190-2

In this article [1], there was a copy pasting error of the PCR image of the representative data provided in Figures 1 and 2. The corrected figures are published with this erratum (Figs. 1 and 2).

**Fig. 1:**
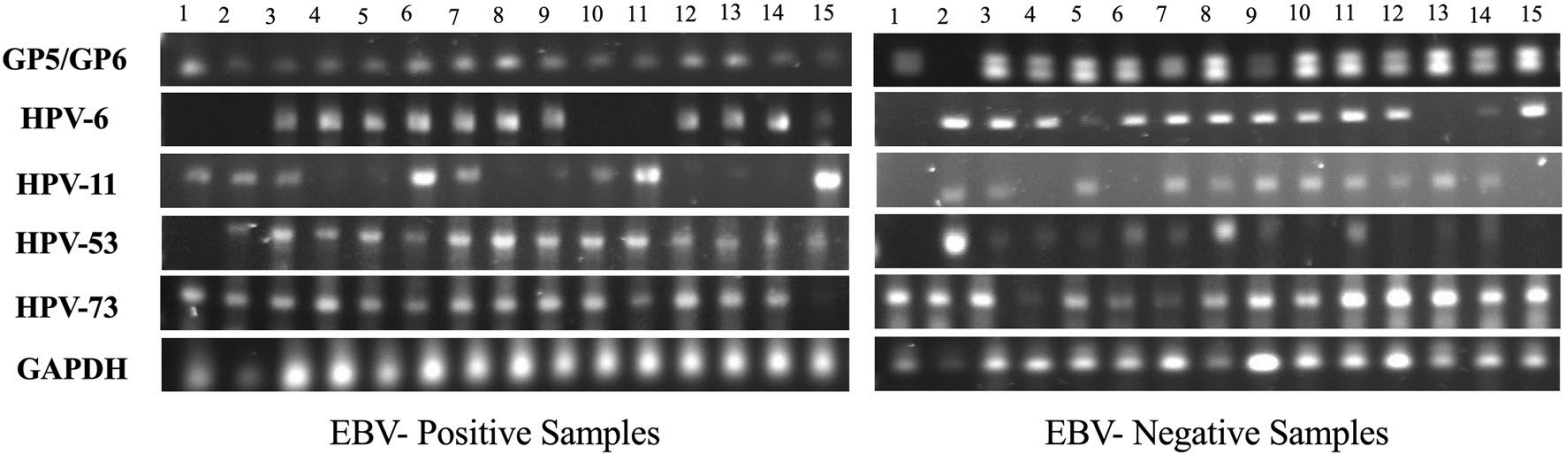
Representative PCR for low-risk HPV-subtypes in 15 different EBV-positive and EBV-negative healthy blood donors

**Fig. 2:**
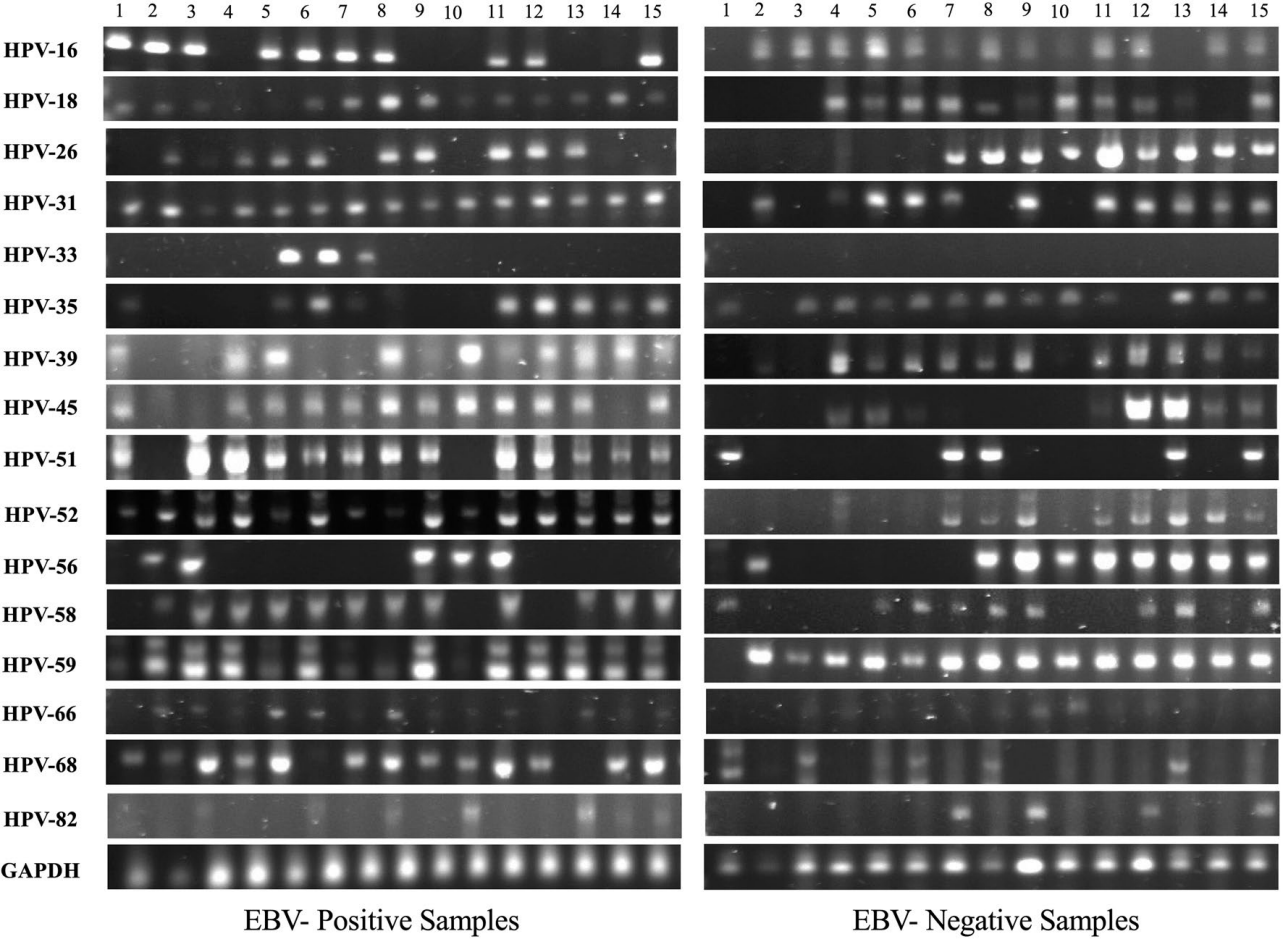
Representative PCR for high-risk HPV-subtypes in 15 different EBV-positive and EBV-negative samples of healthy blood donors
